# Soluble Toll-Like Receptors 2 and 4 in Cerebrospinal Fluid of Patients with Acute Hydrocephalus following Aneurysmal Subarachnoid Haemorrhage

**DOI:** 10.1371/journal.pone.0156171

**Published:** 2016-05-25

**Authors:** Bartosz Sokół, Norbert Wąsik, Roman Jankowski, Marcin Hołysz, Barbara Więckowska, Paweł Jagodziński

**Affiliations:** 1 Department of Neurosurgery, Poznan University of Medical Sciences, Poznan, Poland; 2 Department of Biochemistry and Molecular Biology, Poznan University of Medical Sciences, Poznan, Poland; 3 Department of Computer Science and Statistics, Poznan University of Medical Sciences, Poznan, Poland; Heinrich-Heine University, GERMANY

## Abstract

**Background:**

Toll-like receptor (TLR) signalling begins early in subarachnoid haemorrhage (SAH), and plays a key role in inflammation following cerebral aneurysm rupture. Available studies suggest significance of endogenous first-line blockers of a TLR pathway—soluble TLR2 and 4.

**Methods:**

Eighteen patients with SAH and acute hydrocephalus underwent endovascular coiling and ventriculostomy; sTLR2 and 4 levels were assayed in cerebrospinal fluid (CSF) collected on post-SAH days 0–3, 5, and 10–12. Release kinetics were defined. CSF levels of sTLR2 and 4 were compared with a control group and correlated with the clinical status on admission, the findings on imaging, the degree of systemic inflammation and the outcome following treatment.

**Results:**

None of study group showed detectable levels of sTLR2 and 4 on post-SAH day 0–3. 13 patients showed increased levels in subsequent samples. In five SAH patients sTLR2 and 4 levels remained undetectable; no distinctive features of this group were found. On post-SAH day 5 the strongest correlation was found between sTLR2 level and haemoglobin level on admission (cc = -0.498, P = 0.037). On post-SAH day 10–12 the strongest correlation was revealed between sTLR2 and treatment outcome (cc = -0.501, P = 0.076). Remaining correlations with treatment outcome, status at admission, imaging findings and inflammatory markers on post-SAH day 5 and 10–12 were negligible or low (-0.5 ≤ cc ≤ 0.5).

**Conclusions:**

In the majority of cases, rupture of a cerebral aneurysm leads to delayed release of soluble TLR forms into CSF. sTLR2 and 4 seem to have minor role in human post-SAH inflammation due to delayed release kinetics and low levels of these protein.

## Introduction

Toll-like receptors (TLRs) play a key role in innate immunity. These abundantly expressed receptors recognise pathogen-associated molecular patterns (PAMPs) and endogenous danger-associated molecular patterns (DAMPs). Infectious and non-infectious triggers share a downstream TLR signalling pathway leading to inflammatory gene expression in the target cells [1]. Tight regulation of TLR signalling is crucial to prevent a hyperinflammatory response. Soluble forms of TLRs (sTLRs) found in body fluids are considered to be a first-line blockade in TLR signalling. Actively released extracellular domains of TLR 2 and 4 behave as a decoy receptors. They are capable of ligand (PAMPs and DAMPs) binding, but do not transmit the signal downstream [2]. Elevated serum levels of sTLR4 are associated with infection, predominately with bacterial, while decreased levels of sTLR2 are associated with autoimmune diseases [3–5].

Delayed cerebral ischaemia (DCI) is the major cause of morbidity and mortality after aneurysmal subarachnoid haemorrhage (SAH). In recent years, the key role of a arterial spasm in the pathogenesis of DCI has increasingly been called into question [6]. Among the counter arguments are the disappointing outcomes of the CONSCIOUS-2 trial, which seem to negate such an association, and suggested looking beyond vasospasm [7]. This led to the introduction of the term "early brain injury" (EBI) by Kusaka et al. in 2004 [8]. EBI represents the pathophysiological events occurring during the first 72 hours following aneurysm rupture. Excessive inflammatory reaction leading to detrimental effects is one of the component of EBI [9]. Hence the investigation of the role of soluble TLRs as endogenous blockers in a key inflammatory pathway may prove rewarding.

## Materials and Methods

### Ethics, consent, and permissions

The study was performed in accordance with the Declaration of Helsinki and was approved by the Poznan University of Medical Sciences' bioethics committee (approval no. 983/13). All participants or their families provided written informed consent. Two physicians (anaesthesiologist and neurosurgeon) independently assessed level of patient's consciousness by Glasgow coma scale (GCS) at admission and daily. Patients with GCS score of 14 and lower were qualified as incapable of giving informed consent, therefore written consent was obtained from the next of kin on the behalf of participants. Patients with GCS score of 15 (and 14 in case of eye opening score of 3) underwent decision-making capacity assessment with probing questions [10]. In case of failure, the next of kin provided surrogate consent.

### Study population

Between December 2013 and June 2015, 152 patients with aneurysmal SAH, confirmed by computerised tomography (CT) and digital subtraction angiography, were admitted to the neurosurgical ward. Inclusion criteria for the study were early (< 24 hour post rupture) endovascular occlusion of the aneurysm and acute hydrocephalus (HCP) treated with external ventricular drainage (EVD) before 48 hours post rupture. HCP was diagnosed on the basis of the original CT findings (relative bicaudate index of more than 1 or focal dilation of ventricular system due to obstruction). Patients with decreased consciousness and diagnosed HCP or high likelihood of HCP development (thick intraventricular blood clot) underwent cerebrospinal fluid (CSF) diversion. Due to high risk of brain tissue shift (13/18 patients had intraparenchymal haematoma) and suspected obstructive aetiology of HCP (18/18 patients had intraventricular blood) EVD was preferred. Exclusion criteria were age below 18, pregnancy, history of central nervous system (CNS) disease (meningitis, stroke), active infection of CNS and active systemic disease (diabetes mellitus, rheumatoid arthritis, malignancy, cirrhosis, renal failure). To rule out potential impact of a surgery-related trauma on a CSF homoeostasis, patients who underwent clipping were also excluded. This left a total of eighteen patients in the study group. The control group consisted of eight patients with minor neurological disorders (e.g. tension headache), where CSF was obtained during diagnostic lumbar puncture (LP). The patients with abnormal CSF results, elevated C-reactive protein (CRP) level and white blood cell (WBC) count on the day of LP were not enrolled. The exclusion criteria were the same as for the study group.

### Management protocol

On admission the clinical status was assessed using the Hunt & Hess scale (HH), World Federation of Neurosurgical Societies scale (WFNS), and GCS. After aneurysm occlusion and EVD insertion a CT scan of the head was carried out on post-SAH day 2–3 to assess any procedure related brain injury. All patients received continuous intravenous infusion of nimodipine for at least 10 days, hypotension was avoided using vasopressors and euvolemia was maintained. DCI in our study was defined as neurological impairment (such as hemiparesis, aphasia, apraxia, hemianopsia, or neglect) or a decrease of at least 2 points on the GCS [either on the total score or one of its individual components (eye, motor on either side, verbal)]. Any such deterioration should last for at least one hour, not be apparent immediately after aneurysmal occlusion, and not attributable to non-neurological causes [11]. Every patient diagnosed with DCI was treated with induced hypertension (20–30% rise of mean arterial pressure above baseline). Cerebral infarction due to DCI was defined as presence of cerebral infarction on CT scan of the brain within 6 weeks after SAH, not present on the post coiling/EVD CT scan in patients diagnosed with DCI. Treatment outcome was assessed at three months using the Glasgow outcome scale (GOS).

### Sample collection and assays

Haemoglobin (Hgb), CRP level and WBC count were assessed daily. Automatic analysers XT 2000i (Sysmex, Japan), Cobas 6000 (Roche diagnostic, USA) and ACL TOP 500 (Instrumentation Laboratory, Italy) were used for measurements. CSF samples were collected from the EVD at three time points, on post-SAH day 0–3, 5, and 10–12. The final sample was not obtained in five cases, on account of early EVD removal. CSF cell count was checked at least twice per patient and CSF culture was ordered at least once on post-SAH day 10–12. Each CSF sample was centrifugated, and stored at -80°C until assayed. ELISA commercial kits SEA753Hu and SEA663Hu (USCN Life Science, Wuhan, China) with a lower detection limit of 0.117 ng/ml and 0.133 ng/ml respectively, were used to assess sTLR2 and 4 concentrations. Values below the detection limit were extrapolated using ELISA kit standard curves.

### Statistical analysis

Values for normally distributed numerical data have been expressed as mean and standard deviations; for ordinal or non-normally distributed numerical data as median and interquartile range, and for categorical data as counts and percentages. Since the sTLR2 and 4 levels do not show normal distribution they are presented as median and interquartile range. The normality of data distribution was assessed using the Shapiro-Wilk test. The correlations were assessed by Spearman’s exact test and degree of correlation was described 'according to a rule of thumb': -0.3 ≤ correlation coefficient (cc) ≤ 0.3 as “negligible correlation”; 0.3 < cc ≤ 0.5 (-0.5 ≤ cc < -0.3) as “low correlation”; 0.5 < cc ≤ 0.7 (-0.7 ≤ cc < -0.5) as “moderate correlation”; 0.7 < cc ≤ 0.9 (-0.9 ≤ cc < -0.7) as “high correlation”; cc < 0.9 ≤ 1.0 (-1.0 ≤ cc < -0.9) as “very high correlation” [12]. A value of P < 0.05 was considered statistically significant when comparing. Data were analyzed using STATISTICA 10 (Stat Soft Inc., Tulsa, OK, USA) and StatXact (Cytel Inc., Cambridge, MA, USA).

## Results

**Fig 1:**
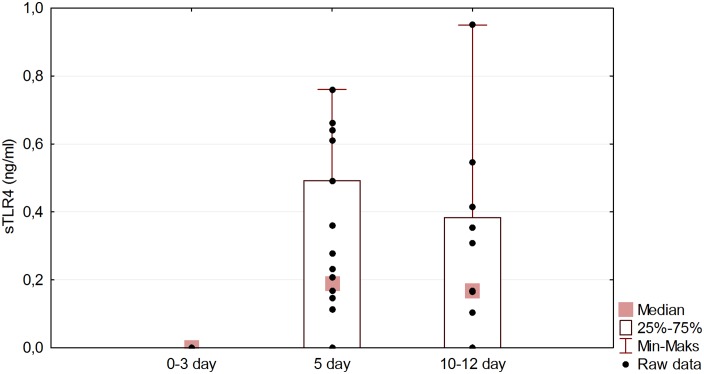
sTLR4 level changes in time in study group. Friedman exact test (P < 0.001) with Conover–Iman post hoc indicates significant difference between post-SAH day 0–3 and post-SAH day 5 (P < 0.001) and also between post-SAH day 0–3 and post-SAH day 10–12 (P < 0.001). Page exact test revealed significant uptrend in sTLR4 level in time (P < 0.01).

**Fig 2:**
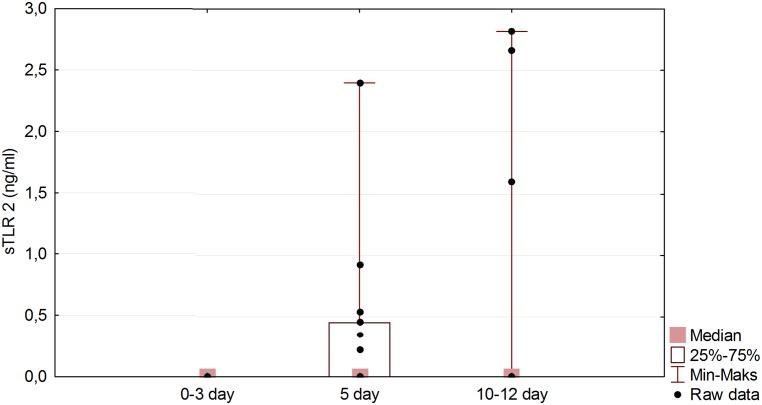
sTLR2 level changes in time in study group. Friedman exact test (P < 0.05) with Conover–Iman post hoc indicates significant difference between post-SAH day 0–3 and post-SAH day 5 (P < 0.01).

The details of the eighteen patients in the study group are shown in Table 1 and S1 dataset. This group did not differ significantly from the eight control subjects in respect of age and sex (P values of 0.362 and 0.382 respectively). sTLR2 and 4 were not detected in any of the control CSF samples. The initial samples (day 0–3) in the study group also showed undetectable levels, but subsequent samples exhibited delayed release. The CSF on day 5 showed sTLR2 in one case, sTLR4 in 6 cases, both proteins in 6 cases and neither in 5 cases. The thirteen samples available from days 10–12 showed a variety of changes compared with those from day 5; there was a fall in the levels of the proteins in 6 cases, a rise in levels in 3 cases, a fall in one and a rise in the other in 2 cases and no change in 2 further cases. There was a significant statistical difference between sTLR4 levels for the first and second samples (P < 0.001), and for the first and third samples (P < 0.001), confirmed by the Friedman exact test with Conover-Iman post hoc. The Page exact test revealed a significant uptrend in the sTLR4 level with time (P < 0.01) (Fig 1). The sTLR2 also showed a significant rise between the first and second samples (P < 0.01) (Fig 2). Correlation data is presented in Table 2. The highest correlation on post-SAH day 5 was found between sTLR2 level and Hgb level on admission (cc = -0.498, P = 0.037). sTLR2 or sTLR4 levels show negligible correlations with treatment outcome (cc = -0.078, P = 0.758 and cc = -0.053, P = 0.834 respectively) and negligible or low correlations with other parameters monitored in our study. In CSF from post-SAH day 10–12 highest correlation was revealed between sTLR2 level and treatment outcome (cc = -0.501, P = 0.076). Remaining parameters presented negligible or low correlation with sTLRs levels on post-SAH day 10–12. The outcome of the study group was highly correlated with the clinical status on admission assessed by WFNS scale (cc = 0.846, P < 0.001), HH scale (cc = 0.729, P < 0.001), and GCS scale (cc = 0.801, P < 0.001). In five SAH patients sTLR2 and 4 were not found in any CSF sample, (although in three of these, the third sample was not available). These five patients showed no statistical difference with regard to treatment outcome or any other parameter monitored in this study when compared with the remainder of the group (Table 3).

**Table 1:** Study group patients’ characteristic.

Male	13 (72%)		
Age (years)	56.78 ± 17.7		
Aneurysm location			
Middle cerebral artery	4 (22%)		
Anterior cerebral artery	4 (22%)		
Anterior communicating artery	7 (39%)		
Basilar artery	2 (11%)		
Posterior cerebral artery	1 (6%)		
Aneurysmal size (mm)	4.62 ± 1.7		
Cerebral infarction due to DCI on CT	13 (72%)		
Intracerebral haemorrhage on CT	13 (72%)		
Intraventricular blood on CT	18 (100%)		
Fisher CT score	4 (4–4)		
Modified Fisher CT score	4 (2–4)		
WFNS score on admission	4 (3–5)		
HH score on admission	4 (4–5)		
GCS on admission	9 (5–12)		
	On admission	Post-SAH day 5	Post-SAH day 10–12
CRP level (mg/l)	93.60 ± 86.4	155.80 ± 112.9	75.77 ± 79.0
WBC count (10^6^/mm^3^)	13.73 ± 5,8	11.54 ± 5.2	14.23 ± 6.7
Hgb level (mg/dl)	12.11 ± 2.8	11.66 ± 1.9	10.92 ± 1.7
sTLR2 (ng/ml)	0 (0–0)	0 (0–0.454)	0 (0–0)
sTLR4 (ng/ml)	0 (0–0)	0.189 (0–0.492)	0.167 (0–0.384)
Treatment outcome (GOS at 3 months)			
low disability (score of 5)	5 (28%)		
moderate disability (score of 4)	2 (11%)		
severe disability (score of 3)	2 (11%)		
persistent vegetative state (score of 2)	2 (11%)		
death (score of 1)	7 (39%)		

Values are presented as mean and standard deviations if normally distributed numerical data; median and interquartile range if ordinal data or non-normally distributed numerical data; counts and percentage if categorical data. Abbreviations: DCI = delayed cerebral ischaemia; CT = computed tomography; WFNS = World Federation of Neurosurgical Societies scale; HH = Hunt and Hess scale; GCS = Glasgow Coma Scale; CRP = C-reactive protein; WBC = white blood cell; Hgb = plasma haemoglobin; sTLR = soluble Toll-like receptor; GOS = Glasgow outcome scale.

**Table 2:** Correlation between monitored parameters, treatment outcome, sTLR2 and 4 CSF levels on post-SAH days 5 and 10–12.

	GOS at 3 months		sTLR2 level on post-SAH day 5		sTLR4 level on post-SAH day 5		sTLR2 level on post-SAH day 10–12		sTLR4 level on post-SAH day 10–12	
Monitored parameters	cc	P value	cc	P value	Cc	P value	cc	P value	cc	P value
WFNS on admission	-0.846	<0.001	0.051	0.840	0.267	0.281	0.276	0.364	0.148	0.639
HH on admission	-0.729	0.001	0.140	0.580	0.481	0.045	0.455	0.150	0.366	0.239
GCS on admission	0.801	<0.001	-0.130	0.606	-0.289	0.242	-0.176	0.566	-0.018	0.957
Modified Fisher CT score	-0.119	0.635	-0.228	0.359	-0.148	0.551	0.167	0.653	0.085	0.799
CRP level	-0.252	0.311	-0.170	0.498	0.054	0.832	0.059	0.854	0.011	0,978
WBC count	-0.661	0.004	0.018	0.948	-0.103	0.682	0.260	0.397	0.470	0,125
Hgb level on admission	-0.040	0.874	-0.498	0.037	-0.016	0.951	0.035	0.913	0.071	0.824
GOS at 3 months	-	-	-0.078	-0.758	-0.053	-0.834	-0.501	0.076	-0.252	0.422

Abbreviations: sTLR = soluble Toll-like receptor; cc = correlation coefficient; WFNS = World Federation of Neurosurgical Societies scale; HH = Hunt and Hess scale; GCS = Glasgow coma scale; CRP = C-reactive protein; WBC = White blood cell; Hgb = plasma haemoglobin; GOS = Glasgow outcome scale

**Table 3:** Comparison of patients with present and absent sTLRs in all three CSF samples.

	sTLR2 and 4 present in CSF	sTLR2 and 4 absent in CSF	P value
Number of patients	13	5	
Male	9 (69%)	4 (80%)	1.00
Age (years)	56.69 ± 19.9	57.00 ± 12.0	.975
Aneurysm location			.467
Middle cerebral artery	3 (23%)	1 (20%)	
Anterior cerebral artery	3 (23%)	1 (20%)	
Anterior communicating artery	6 (46%)	1 (20%)	
Basilar artery	1 (8%)	1 (20%)	
Posterior cerebral artery	0	1 (20%)	
Aneurysmal size (mm)	4.2 ± 1.3	5.6 ± 2.3	.977
Cerebral infarction due to DCI on CT	10 (77%)	3 (60%)	.583
Intracerebral haemorrhage on CT	11 (84%)	2 (40%)	.099
Intraventricular blood on CT	13 (100%)	13 (100%)	1.00
Modified Fisher CT score	4 (2–4)	4 (2–4)	.909
WFNS score on admission	4 (3–5)	4 (4–5)	.958
HH score on admission	4 (4–5)	4 (4–4)	.250
GCS on admission	9 (5–12)	9 (5–9)	1.00
CRP level (mg/l)	89.1 ± 88.2	97.5 ± 82.6	.844
WBC count (10^6^/mm^3^)	12.3 ± 4.1	15.8 ± 9.5	.280
Hgb level (mg/dl)	11.4 ± 2.6	13.7 ± 2.6	.106
Treatment outcome (GOS at 3 months)	2 (1–5)	3 (1–4)	.918

Values are presented as mean and standard deviations if normally distributed numerical data; median and interquartile range if ordinal data or non-normally distributed numerical data; counts and percentage if categorical data. Abbreviations: sTLR = soluble Toll-like receptor; DCI = delayed cerebral ischaemia; CT = computed tomography; WFNS = World Federation of Neurosurgical Societies scale; HH = Hunt and Hess scale; GCS = Glasgow Coma Scale; CRP = C-reactive protein; WBC = white blood cell; Hgb = plasma haemoglobin; GOS = Glasgow outcome scale.

## Discussion

Our study revealed that, in the majority of cases, aneurysm rupture leads to the release of soluble TLR2 and 4 forms into the CSF. We are not aware of any previous reports of soluble TLRs appearing in CSF, therefore potential origin, release pattern and significance of intrathecal sTLRs are discussed.

### Origin of sTLR2 and 4

ELISA kits used in our study lack the ability to differentiate between strictly soluble and membrane forms of TLRs. We believe that the sTLR values presented consist of (I) soluble TLRs released by immune cells as a TLR signalling blockade, and (II) membrane TLRs released incidentally as a result of necrosis and cell membrane fragmentation. This raises the question as to which form dominates in CSF. In studies using identical ELISA kits, membrane forms of TLRs have been ignored in discussions about the origins of the assayed sTLR [3,4,13,14]. TLRs are abundantly expressed in CNS and their mRNA is upregulated in the brain in SAH and ischaemic models [15–17]. Most animal studies show that both necrosis and apoptosis are modes of cell death following SAH, but necrosis is not considered to be a major factor [18,19]. On this basis, and in line with other authors, we have assumed that the membrane TLR form makes only a minor contribution to the measured sTLRs values.

**Fig 3:**
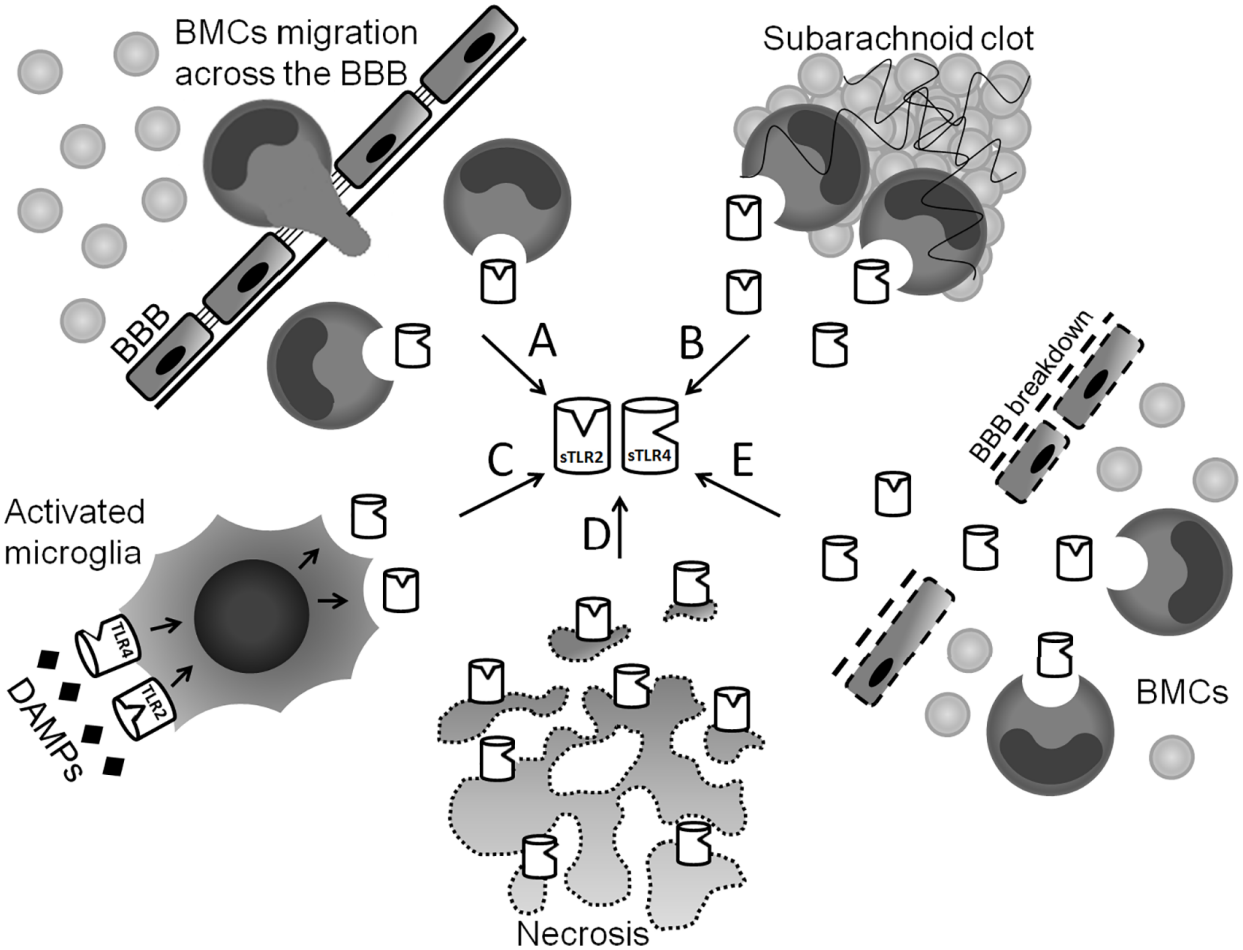
Possible sources of sTLR2 and 4 in CSF. (A) Migration of activated BMCs across the BBB during SAH and active release of sTLRs into CSF. (B) BMCs trapped in subarachnoid clot actively release sTLRs into CSF. (C) DAMPs bind to TLRs on microglia surface and trigger TLR signalling followed by active release of sTLRs into CSF. (D) Membrane TLRs released incidentally into CSF as a result of necrosis and cell membrane fragmentation. (E) BMCs release sTLRs peripherally into the blood plasma. sTLRs leak through the disrupted by SAH BBB. Abbreviations: CSF = cerebrospinal fluid; sTLR = soluble Toll-like receptor; BMC = blood mononuclear cells; DAMP = danger-associated molecular patterns; BBB = blood-brain barrier.

Lack of sTLR2 and 4 in CSF from the control group is consistent with the inflammatory-dependent nature of these receptors. Physiologically, sTLR2 and 4 have been shown to be present in blood plasma, saliva, breast milk and amniotic fluid [3,20–22]. As mentioned above, presence of sTLR2 and 4 in CSF may be a consequence of active production (intrathecal or peripheral) and incidental release (intrathecal necrosis) (Fig 3). The only established source of soluble TLR forms are blood mononuclear cells (BMCs) [3]. Their intrathecal appearance may be due to some SAH-related migration through the blood-brain barrier (BBB), or that BMCs are simply released from the subarachnoid blood clot into the CSF [23]. An alternative local sTLR2 and 4 source may be the microglia. These resident CNS macrophages express both TLR2 and 4 on their surfaces, and are activated during SAH [24,25]. BBB breakdown observed in SAH allows migration of blood-derived proteins into the CNS. Potentially, sTLR2 and 4 circulating in blood may reach the CSF by this route [26]. Although it is suspected to be a minor contribution, incidental release of membrane TLRs due to cell necrosis within the CNS should not be neglected.

### Release kinetics of sTLR2 and 4

In our study, sTLR2 and 4 level changes followed a specific pattern; neither can be detected on post-SAH day 0–3, but then increase with time in the majority of cases. By contrast, plasma concentrations of sTLR2, sTLR4, and IL-1Ra peak in 2–4 hours following lipopolysaccharide (LPS) administration in all patients [3]. Delayed sTLR2 and 4 appearance in CSF denies necrotic origin of sTLR2 and 4, since some cell death might be expected at an early stage following aneurysm rupture, and this would lead to the presence of sTLR in the early samples. Conversely, delayed kinetics may reflect the time required for the migration of activated BMCs to the CNS before local sTLRs production can be initiated. Alternatively, unlike LPS, DAMPs may trigger a prolonged sTLR2 and 4 production pathway in migrated BMCs or microglia. As mentioned above, current reports describe only a rapid (peak in 2–4 hours) release of sTLR2 and 4 to blood plasma after stimulation with LPS (an example of a PAMP).

### Potential significance

Soluble forms of TLRs function as the first line of blockade in TLR signalling [2]. Abundant expression of TLRs in CNS (especially on microglia) can lead to a massive inflammatory reaction [24]. Reported observations of (I) early release of DAMPs during SAH, (II) upregulation of downstream mediators and TLRs in brain tissue and (III) increase of final protein products in CSF and brain tissue, indicate both activation of TLR signalling and failure of anti-inflammatory mechanisms [16,27–31]. Attenuation of SAH associated neuronal apoptosis in TLR4 depleted mice and alleviation of EBI in rats administered exogenous TLR signalling blocker, prove both the substantial role of TLRs in experimental SAH, and the therapeutic potential of TLR signalling blockers [32,33]. Revealed in our study negligible to moderate correlations between sTLRs and patients’ condition seem to lessen significance of sTLRs in human SAH. We found this observation unexpected, thus it is discussed below. Firstly, CSF concentrations of soluble TLRs are low in comparison with other body fluids. Blood plasma from healthy volunteers has 3–8 times the concentration of sTLR2, while experimental endotoxemia leads to plasma concentrations of sTLR2 and 4 which are 10–100 times greater [3,4]. Secondly, sTLR2 and 4 were absent from CSF at 0–3 days and thus cannot influence TLRs signalling, which peaks 2–6 hours after aneurysm rupture [16]. Thirdly, in vast majority we have found negligible to low correlation between sTLR2 and 4 levels and the severity of inflammation (defined by WBC, CRP level), clinical status on admission and treatment outcome. Furthermore, complete absence of soluble TLRs in CSF of 5 patients did not affect their treatment outcome, or lead to any differences in the monitored parameters. The results of our study suggest that delayed release kinetics and low concentrations of soluble TLRs (while DAMPs levels are elevated), might be responsible for low overall correlations with clinical status.

### Limitations of the study

EVD infection is a potential bias. Microbial CNS inflammation would be likely to lead to profound alterations in sTLRs levels. Since all of these patients had an intraventricular drain, infection was a distinct possibility. However, no drain was removed early as a result of infection, and none showed evidence of infection on removal. Nearly all patients received antibiotics for other unrelated infections while the drains were in situ what could be a bias. It is recognised that this group of patients does not represent the full spectrum of aneurysmal SAH, which limits the range of possible conclusions. The study protocol restricted number of enrolled patients and determined their poor admission status. However the basic correlation between presenting status and clinical outcomes does not suggest any other unusual feature for this group. On the basis of available literature, we assumed robust activation of TLR signalling in our patients, and did not measure levels of TLR pathway products to confirm this [16,32,34].

### Future directions

We consider that confirmation of sTLR release into CSF following SAH should encourage further studies in view of the possible role in delayed brain injury. In order to investigate this further, we would suggest a larger series of all grade SAH patients, with stricter monitoring of potential CNS infection and antibiotic intake. sTLR levels should be analysed together with other elements of the TLR signalling pathway in both blood and CSF.

## Conclusions

In the majority of cases, rupture of a cerebral aneurysm leads to delayed release of soluble TLR forms into CSF. sTLR2 and 4 seem to have minor role in human post-SAH inflammation due to delayed release kinetics and low levels of these protein.

## Supporting Information

S1 Dataset Raw Data. (XLSX)
