# Correction: Soluble Toll-Like Receptors 2 and 4 in Cerebrospinal Fluid of Patients with Acute Hydrocephalus following Aneurysmal Subarachnoid Haemorrhage

**DOI:** 10.1371/journal.pone.0157853

**Published:** 2016-06-14

**Authors:** Bartosz Sokół, Norbert Wąsik, Roman Jankowski, Marcin Hołysz, Barbara Więckowska, Paweł Piotr Jagodziński

The last author’s name is incomplete. The complete name is: Paweł Piotr Jagodziński. The correct citation is: Sokół B, Wąsik N, Jankowski R, Hołysz M, Więckowska B, Jagodziński PP (2016) Soluble Toll-Like Receptors 2 and 4 in Cerebrospinal Fluid of Patients with Acute Hydrocephalus following Aneurysmal Subarachnoid Haemorrhage. PLoS ONE 11(5): e0156171. doi:10.1371/journal.pone.0156171.
